# Cardiac Troponin as a Prognostic Indicator for Major Adverse Cardiac Events in Non-Cardiac Surgery: A Narrative Review

**DOI:** 10.3390/diagnostics15091061

**Published:** 2025-04-22

**Authors:** Syarifah Noor Nazihah Sayed Masri, Fadzwani Basri, Siti Nadzrah Yunus, Saw Kian Cheah

**Affiliations:** 1 Department of Anaesthesiology & Intensive Care, Faculty of Medicine, Hospital Canselor Tuanku Muhriz, Universiti Kebangsaan Malaysia, Kuala Lumpur 56000, Malaysia; skii_cheah@yahoo.com; 2 Department of Anaesthesiology & Intensive Care, Hospital Kuala Lumpur, Kuala Lumpur 50586, Malaysia; fadzwani_basri@moh.gov.my; 3 Department of Anaesthesiology & Intensive Care, Faculty of Medicine, University of Malaya, Kuala Lumpur 50603, Malaysia; siti.nadzrah@ummc.edu.my

**Keywords:** cardiac troponin, myocardial injury following non-cardiac surgery (MINS), major adverse cardiovascular events (MACEs), highly sensitive troponin

## Abstract

A major adverse cardiac event (MACE) following non-cardiac surgery encompasses critical postoperative cardiovascular complications such as myocardial infarction or injury, cardiac arrest, or stroke that are associated with increased perioperative morbidity, mortality, and healthcare resource utilisation. Cardiac troponin (cTn), particularly high-sensitivity cardiac troponin (hs-cTn), has emerged as a key biomarker for prediction of MACE. Despite its recognised utility, there is no consensus on how cTn levels should be used for standardised postoperative surveillance. Interpretation of the cTn levels may vary depending on sex-specific reference values and baseline comorbidities such as chronic kidney disease, sepsis, critical illness, and non-ischaemic conditions. The balance between cost-effectiveness and clinical benefit in implementing universal versus targeted postoperative hs-cTn screening remains to be fully explored. This review examines the prognostic value of cardiac troponin (cTn) levels in predicting major adverse cardiovascular events (MACEs) in patients undergoing non-cardiac surgery, with a focus on perioperative cTn elevations—particularly those associated with myocardial injury after non-cardiac surgery (MINS)—as potential early indicators of increased cardiovascular risk.

## 1. Introduction

Major adverse cardiac events (MACE) following non-cardiac surgery encompass a vast spectrum of clinical conditions, including acute myocardial infarction, acute heart failure, haemodynamically relevant arrhythmia, myocardial injury, cerebrovascular accident, and death, which adversely impact patient recovery and survival [1]. Their incidence varies between 12 and 25% postoperatively [1,2]. Advanced age over 65 years old; comorbidities such as hypertension, diabetes mellitus, and chronic heart failure; and surgical factors like emergency and high-risk surgeries are all associated with an increased risk of major adverse cardiovascular events (MACEs) [3,4]. Beyond comorbidities, frailty has been identified as an additional factor that further elevates the risk of MACE [5]. The pathophysiology of MACE is multifaceted, encompassing pre-existing risk factors, surgical stress response, increased sympathetic activity, and systemic inflammatory response, resulting in an imbalance between myocardial oxygen supply and demand [3]. The influence of MACE is substantial, impacting both short-term and long-term outcomes post-surgery. Patients with MACE face a threefold increased risk of mortality within one year compared to those without such episodes [6]. The occurrence of MACE is associated with increased disability, impacting patients’ quality of life and functional capacity long after the surgical procedure [6]. Conversely, postoperative MACE substantially contributes to higher health care costs and resource utilization [7]. The financial implications of MACE extend beyond direct hospital expenses, as patients with MACE frequently necessitate additional postoperative hospital visits and experience functional impairment and reduced quality of life [8].

Myocardial injury after non-cardiac surgery (MINS) is defined as an elevation in cardiac troponin levels following non-cardiac surgery, representing a manifestation of myocardial ischaemia that occurs within 30 days post-procedure [9]. Diagnosis of MINS does not require overt ischaemic symptoms such as chest pain or shortness of breath, nor does it necessitate electrocardiographic changes [10]. Instead, MINS is identified when at least one postoperative troponin level exceeds the 99th percentile of the upper reference limit for the specific assay used [9]. Therefore, routine cardiac troponin monitoring is essential for accurate diagnosis. The pathophysiology of MINS involves a mismatch between myocardial oxygen supply and demand, often worsened by surgical stress responses such as tachycardia, hypertension, or hypotension. This imbalance leads to myocardial ischaemia, resulting in elevated cardiac troponin levels [2]. Additionally, MINS can be triggered by coronary plaque rupture or thrombosis, although coronary artery disease is not always the cause. Notably, only a small percentage of MINS patients demonstrate evidence of coronary thrombus on cardiac catheterization [11]. Several risk factors have been identified for the occurrence of MINS during the perioperative period. Factors include advanced age, male gender, and comorbid conditions such as cardiovascular disease, cerebrovascular disease, diabetes mellitus, peripheral artery disease, aortic disease, and renal insufficiency. Operative variables, including duration, type, and extent of the procedure and intraoperative hypotension contribute to the increase myocardial oxygen demand [12]. Anaemia also has been extensively found to have a correlation with MINS [13,14].

The elevation of cardiac troponin primarily mediates the relationship between MACE and MINS. Studies have consistently shown that pre and postoperative troponin elevations are strongly associated with an increased risk of MACE [15,16]. An elevation in postoperative troponin levels has been demonstrated to predict death at 30 days and 1 year postoperatively and is associated with an increased incidence of major adverse cardiovascular events (MACEs) at similar intervals. These findings underscore the importance of perioperative troponin surveillance due to its prognostic capacity to predict MACEs and postoperative mortality following noncardiac surgery.

There are various scoring systems assessing the risk of major adverse cardiac events (MACEs) following non-cardiac surgery, such as the Revised Cardiac Risk Index (RCRI), American College of Surgeons National Surgical Quality Improvement Program (ACS NSQIP), and Surgical Outcome Risk Tool (SORT) [17,18]. Biomarkers such as N-terminal pro-B-type natriuretic peptide (NT-proBNP) and cardiac troponin can provide an objective, real-time assessment through which to evaluate and predict mortality in high-risk surgical patients for non-cardiac surgery, and they can aid in identifying patients who require the additional perioperative care and monitoring [15,19]. Therefore, our review aims to evaluate the utility of cardiac troponin levels in predicting myocardial injury in non-cardiac surgery (MINS) and its association with major adverse cardiovascular events (MACE).

## 2. Perioperative Cardiac Troponin Elevation and Its Causes

**Figure 1:**
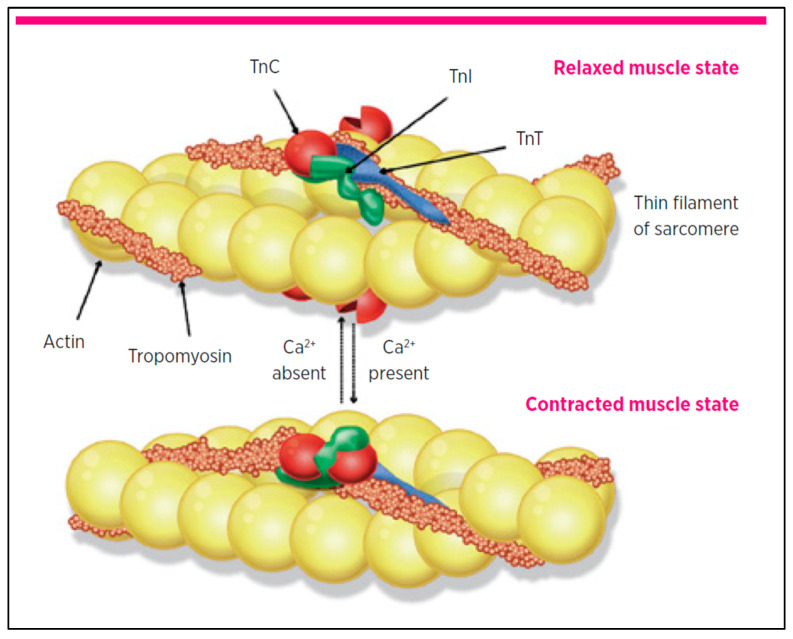
Schematic representation of the cardiac troponin complex’s role in muscle contraction. Cardiac muscle showing location of cardiac troponin I (TnI), cardiac troponin T (TnT), and cardiac troponin C (TnC) [20] (adapted with permission).

Troponin is a protein found in the thin filament of skeletal and cardiac muscle, consisting of three subunits: I, T, and C. These subunits work with calcium to regulate muscle contraction (Figure 1) [20]. Troponin T associates the troponin complex with the actin filament, while troponin C is the calcium-binding site. Troponin I inhibits the interaction with myosin heads when calcium ions are insufficient [21]. Troponin I and T are primarily located in the myocardium and are referred to as cardiac troponins (cTnI and cTnT), whereas Troponin C is synthesised in both skeletal and cardiac muscle. Approximately 95% of troponin complexes are situated within the cardiac sarcomere, with the remaining 5% freely present in the cytoplasm [22].

Levels of troponin T and I start increasing 4 to 9 h after acute myocardial infarction. They peak at 12 to 24 h [23]. Levels can remain elevated for up to 14 days, enhancing its utility in detecting silent or delayed presentations of myocardial damage [24]. Thrombosis or plaque rupture may be the primary cause of coronary occlusion, which can result in myocardial injury. Secondary causes, also referred to as type 2 or non-ischaemic myocardial injury, are thought to be caused by an imbalance between the coronary oxygen supply and demand, which can happen during the perioperative phase. In the initial 3–4 h after myocardial injury, elevated levels of cardiac troponin I and T originate from the cytoplasm. Subsequently, these levels progressively rise over the next 4–10 days due to the continued release of structural troponin complexes from ischaemic cardiac myocytes and the degeneration of myofibrils [22]. In the perioperative period, the patient is subjected to surgical stress and sympathetic and inflammatory response. The magnitude of catecholamine release, especially during a major surgery, will result in tachycardia, hence reducing diastolic coronary filling time over a state of increasing myocardial oxygen demand. The effect of this mismatch is particularly concerning in patients with coronary artery disease or limited myocardial structural reserve. Other than that, conditions such as anaemia, persistent hypotension, coronary vasospasm, and hypoxia may worsen the condition [25]. This event is particularly concerning in patients with limited coronary or myocardial structural reserve. Perioperative complications such as pulmonary embolism, sepsis, and kidney injury are also known as non-ischaemic causes of raised troponin [26,27]. Table 1 shows the list of possible factors that may cause raised cardiac troponin during the perioperative period.

Over the past two decades, highly sensitive cardiac troponin (Hs-cTn) has emerged as a highly sensitive biomarker capable of detecting myocardial damage at lower levels compared to traditional troponin assays [22]. The key advantages of hs-cTn include a higher negative predictive value and shorter troponin-blind period, which is typically less than 2–3 h, compared to conventional troponin tests. These benefits enable faster detection of myocardial ischaemia or injury, shorter emergency room stays, and improved cost-effectiveness [28,29].

## 3. Cardiac Troponin as a Predictor for MACE

Serum cardiac troponin, either I or T, is a biomarker of myocardial injury, and has a significant predictive value for major adverse cardiovascular events (MACEs) after non-cardiac surgery [15,16]. Elevated cardiac troponin levels, both preoperatively and postoperatively, are associated with an increased risk of MACE and death. This predictive ability is critical for risk stratification and management of patients undergoing noncardiac surgery.

One systematic review and meta-analysis of 20 studies with a sample size of 13,386 aimed at determining if preoperative cardiac troponin (cTn) predicts short- and long-term poor outcomes and whether perioperative changes in cTn predict short- and long-term adverse outcomes. Adverse outcomes were defined as MACE and/or all-cause mortality. Preoperative cTn was a significant, unadjusted predictor of short-term poor outcomes (OR 4.3, 95% CI 2.9–6.5, *p* < 0.001). However, the author was unable to conduct pooled analyses for unadjusted estimates of perioperative change in cTn as a predictor of long-term adverse outcomes due to an insufficient number of studies. This meta-analysis indicated that preoperative cTn is a predictor of unfavourable outcomes, specifically MACE and/or all-cause death, in adult noncardiac surgery patients; however, the overall prognostic performance of cTn remains uncertain and warrants further exploration [16].

A meta-analysis examining the effect of postoperative troponin elevation on major adverse cardiovascular events (MACE) revealed comparable findings. Ekeloef et al. demonstrated that postoperative troponin elevation serves as a predictor for 30-day mortality, with an odds ratio (OR) of 3.52 [95% confidence interval (CI) 2.21–5.62; I^2^ = 0%] [15], and as an independent predictor for 1-year mortality, with an adjusted OR of 2.53 (95% CI 1.20–5.36; I^2^ = 26%). Postoperative elevation in troponin levels was linked to major adverse cardiac events at 30 days, with an odds ratio of 5.92 (95% CI 1.67–20.96; I^2^ = 86%), and at 1-year post-surgery, the adjusted odds ratio was 3.00 (95% CI 1.43–6.29; I^2^ = 21%) [15].

A prospective observational study involving 2265 patients revealed that those with perioperative myocardial damage, indicated by elevated hs CTnT levels, had an increased risk of acute heart failure, haemodynamically significant arrhythmias, spontaneous myocardial infarction, and mortality within one-year post-surgery. The author also indicated that the susceptible period for patients to experience MACEs following surgery is five months. The study concluded that MACE screening utilising hs-cTnT measures preoperatively (and on postoperative days 1 and 2 for patients identified as high-risk) could improve perioperative care [3].

Based on the studies mentioned above, it can be concluded that pre and postoperative troponin monitoring helps to risk-stratify patients at risk of cardiovascular events after surgery. However, there were variations in MINS incidence across studies, attributed to differences in troponin monitoring time frames, the concurrent evaluation of troponin levels with other clinical indicators or symptoms, and the use of different troponin assays [15,16]. Most of the studies used hs-cTnT or hs-cTnI depending on the center, with both assays demonstrating high diagnostic accuracy for myocardial injury, but hs-cTnI may provide superior prognostic information in specific populations, such as those with renal failure [30]. Troponin C was not used as a predictor as it is not specific to the myocardium.

The international multicenter prospective study (Vascular Events in Noncardiac Surgery Patient Cohort Evaluation) included 21,842 individuals who underwent noncardiac surgery and revealed that 18% exhibited MINS, as evidenced by elevated Hs-cTnT levels [9]. Devereux et al. found that, compared to the non-MINS group (hs-cTnT < 5 ng/L), peak postoperative MINS’s hs-cTnT levels of 20 to <65 ng/L, 65 to <1000 ng/L, and 1000 ng/L or higher had 30-day mortality rates of 3.0%, 9.1%, and 29.6%, respectively as per Table 2. Notably, 93% of the patients diagnosed with MINS in this study were asymptomatic [9]. This highlights the diagnostic challenge of relying only on symptoms and electrocardiogram changes without incorporating troponin biomarker surveillance.

Notably, risk prediction using cardiac troponin extends beyond non-cardiac surgery. Traditionally, the increase in troponin in cardiac surgery is typically overlooked due to the complexities in interpreting the enzyme’s elevation associated with the pathophysiology of the procedure. Dushnowski et al. conducted a prospective analysis on patients undergoing heart valve surgery, demonstrating that baseline NT-proBNP and hs-cTnT effectively predicted postoperative cardiogenic shock, with areas under the receiver operating characteristic curve for the primary endpoint of 0.726 and 0.839, respectively [31]. In this patient population, individuals experiencing postoperative cardiogenic shock necessitating mechanical circulatory support exhibited a considerably increased length of stay, accompanied by a mortality rate of 3.6%. This study indicates the significant impact of cardiac troponin in predicting significant risks following heart valve surgery.

Interestingly, despite being preoperatively classified as low-risk, the patients experienced an elevated postoperative troponin level and, in comparison to the general population, had a higher 1-year mortality risk following surgery [32]. The author concluded that postoperative elevation of cardiac troponin cannot be predicted by risk assessments such as the Revised Cardiac Risk Index or the American College of Surgeons National Surgical Quality Improvement Program. This raises the question of whether detecting myocardial injury after non-cardiac surgery (MINS) postoperatively is critical for improving long-term survival. Table 3 summarizes relevant studies that have examined the prognostic value of troponin in the prediction of MACE.

## 4. Clinical Utility and Guidelines

### 4.1. Preoperative Risk Stratification

An ideal clinical management strategy in the prevention of MACEs involves effective risk stratification that may direct appropriate serology testing for cTn and/or BNP/NT pro-BNP at the preoperative level. The Revised Cardiac Risk Index (RCRI) is a widely used tool for predicting major adverse cardiac events (MACEs), myocardial infarction (MI), and all-cause mortality, offering a reliable means of risk stratification in surgical patients [17]. However, incorporating cardiac biomarkers such as cTn can further enhance its predictive and prognostic value. The addition of troponin to the RCRI significantly improves its ability to predict postoperative cardiac events, with studies showing a moderate increase in the c-statistic (0.14) for MACE prediction and a modest improvement in risk stratification, as reflected by a net reclassification index (NRI) of 0.16. The combination of NT-proBNP and troponin further enhances prediction, yielding a moderate c-statistic increase (0.12) and substantial improvements in NRI [39]. These findings demonstrate that while the RCRI provides valuable prognostic information on its own, integrating cardiac troponin offers incremental predictive and prognostic value, improving the accuracy of risk stratification for MACE, MI, and mortality.

Given the strong evidence supporting the prognostic value of cardiac troponin in predicting major adverse cardiovascular events (MACEs), several medical societies recommend incorporating high-sensitivity cardiac troponin (Hs-cTn) surveillance in patients at increased perioperative risk [40]. The most recent American Heart Association guideline has proposed a detailed stepwise approach to perioperative cardiac assessment that also includes a serology test of cTn and BNP/NT pro-BNP as a fourth-line option at the preoperative stage to justify the patient’s perioperative cardiac risk. An abnormal result in these biomarkers could aid best-practice decisions, including a multidisciplinary discussion that includes palliative care planning [41]. However, the European association reached a consensus on the indication to test either hs Tn T/I or BNP/NT-proBNP or both for intermediate and high non-cardiac surgeries in patients ≥ 65 years old with cardiovascular disease or RCRI ≥ 1 in a routine preoperative cardiac assessment (ESC 2022). Testing cTn or BNP is also a form of objective assessment to support individualised risk prediction, facilitating patient discussions in a shared decision-making process [42]. The Canadian Cardiovascular Society (CCS) recommends performing an electrocardiogram (ECG) and measuring troponin levels daily for 48–72 h after surgery in elderly patients aged ≥ 65 years, those with elevated N-terminal pro-B-type natriuretic (NT-proBNP) levels, and younger patients with significant cardiovascular disease [43].

### 4.2. Algorithmic Approach to Elevated Troponin

A systematic algorithm following a risk identification for MACE is necessary to facilitate the patient care pathway. Preoperative raised cTn or BNP/NT pro-BNP not only confers additional value when added to RCRI but also implies the need for clinical strategies in mitigating the risk of MACE. In a healthy patient, cTn and BNP are present in the circulation in a range of concentrations from about 3 to about 50 ng/L, depending on the patient’s age and gender [44,45]. An increase in cTn from a normal upper limit necessitates high-vigilance monitoring in the perioperative period. Intraoperatively, advanced haemodynamic monitoring for goal-directed therapy should be considered for high-cardiac-risk patients undergoing major non-cardiac surgery. Strict monitoring is required to avoid a reduction in mean arterial pressure to less than 20% from baseline or <60 mmHg for more than 10 min [42,46]. Postoperatively, serial monitoring of the cardiac biomarkers daily for 48 up to 72 h after surgery is recommended [9,43]. Early detection of raised cardiac biomarkers in asymptomatic patients should prompt the clinician to look for causes, which may range from non-cardiac to non-ischaemic and ischaemic cardiac causes. This triggered a basic workup, such as a serial 12-lead ECG recording, to exclude myocardial ischaemia and increase alertness, including raising the threshold of transfusion and fluid and electrolyte management to achieve optimal clinical targets, i.e., Hb > 8 g/dL, potassium level > 4.5 g/dL, and strict fluid balance. Further implications may include an escalation of the level of care for the high-dependency ward or critical care area. Therefore, a further systematic approach in response to abnormal cardiac biomarkers should be discussed at the institutional level depending on the laboratory support and the extent of interventional cardiology services.

### 4.3. Point-of-Care Testing (POCT) for Cardiac Troponin

The feasibility of using high-sensitivity cardiac troponin (hs-cTn) testing in a point-of-care (POC) setting appears quite promising based on several factors such as low running cost, immediate results, and minimal serum sampling [47]. High-sensitivity cardiac troponin (hs-cTn) assays are relatively cost-effective, particularly when using automated platforms or recent POC testing methods [48]. This makes it viable for routine use in both major hospitals and laboratory settings. Given that hs-cTn is highly specific to cardiac injury, its relatively low cost compared to other advanced diagnostic tests for cardiovascular conditions (e.g., imaging, CT scans) contributes to its affordability. The ability to obtain results within 20–30 min is a significant advantage. This rapid turnaround is crucial in acute settings, allowing for quicker clinical decision making, particularly in emergency departments. The ability to perform hs-cTn tests with minimal sample volumes is another advantage. It typically requires a small amount of whole blood, which can be collected via a fingerprick or other minimally invasive techniques [49]. This reduces the discomfort and logistical burden on patients and healthcare providers alike.

The integration of hs-cTn testing, especially in a point-of-care testing format, is highly feasible from both a clinical and economic perspective. Its low cost, rapid result turnaround, minimal sample requirements, and established performance make it an excellent choice for routine cardiac biomarker testing. Furthermore, the reduced need for additional sampling and the ability to provide immediate results align well with the increasing demand for rapid diagnostics in clinical settings. The ongoing evolution of POC technologies could further enhance accessibility, particularly in resource-limited environments.

### 4.4. Therapeutic Implications and Preventive Strategies

Emerging therapeutic evidence further underscores the utility of cardiac troponin monitoring. In a clinical trial, 1754 patients diagnosed with MINS were randomly assigned to receive either the dabigatran or a placebo [50]. Patients in the dabigatran group were treated with a dosage of 110 mg twice daily for up to 2 years. The study results showed that patients in the dabigatran group had a reduced risk of vascular complications, including vascular mortality, myocardial infarction, ischaemic stroke, peripheral arterial thrombosis, amputation, and symptomatic venous thromboembolism, without an additional risk of bleeding. These findings highlight the potential for troponin-guided management strategies to detect MINS and reduce the long-term burden of MACE.

## 5. Challenges and Controversies of Cardiac Troponin

### 5.1. Non-Ischaemic Elevation and Diagnostic Ambiguity

While high-sensitivity cardiac troponin (hs-cTn) is a highly specific marker for myocardial injury in non-cardiac surgery (MINS), its elevation is not exclusive to ischaemic events. It may also be elevated in a variety of non-ischaemic conditions such as atrial fibrillation, stroke, sepsis, and pulmonary embolism, which can lead to false-positive interpretations in the perioperative setting [22,26]. Consequently, clinicians must exercise clinical judgement when interpreting hs-cTn levels, avoiding overdiagnosis of MINS and unnecessary interventions. Differentiating between true myocardial injury and non-ischaemic causes remains a critical diagnostic challenge, particularly in complex perioperative patients.

### 5.2. Sex-Specific Differences in Troponin Thresholds

Another important concern is the underdiagnosis of myocardial injury in women due to sex-based differences in troponin reference ranges. High-sensitivity assays have consistently shown that women generally have lower 99th percentile upper reference limits (URLs) for hs-cTn compared to men. Applying uniform diagnostic thresholds risks missing clinically relevant cardiac events in female patients. In order to improve diagnostic precision, several studies and consensus statements now recommend the adoption of sex-specific 99th percentile values when interpreting hs-cTn results. Incorporating sex-specific thresholds into perioperative protocols could significantly improve the detection of MINS and help reduce disparities in cardiovascular outcomes [51,52].

### 5.3. Chronic Kidney Disease and Elevated Baseline Troponin

Chronic kidney disease (CKD) and critically ill patients generally exhibit elevated baseline levels of Hs-cTn. According to the cross-sectional study conducted within the Chronic Renal Insufficiency Cohort (CRIC), predictors of elevated Hs-cTnT levels include patients with lower estimated glomerular filtration rate (eGFR), increased left ventricular mass, and higher levels of systemic inflammation [53]. Clinicians might erroneously associate elevated Hs-cTn levels in CKD patients undergoing surgery with MINS postoperatively. Hence, it is essential to monitor sequential Hs-cTn levels over time, especially in CKD patients who have undergone surgery, to ensure timely and accurate diagnosis of MINS.

### 5.4. Cost-Effectiveness and Selective Testing Strategies

Even though Hs-CTn is highly sensitive and prognostic, its routine use in perioperative settings is not universally recommended due to unclear cost–benefit ratios. A single-centre prospective cohort study involving approximately 1400 patients showed a calculated incremental cost-effectiveness ratio (ICER) per additionally detected perioperative myocardial injury(PMI) of €425 [54]. These results suggest that systematic PMI screening with hs-cTnT may be cost-effective in the short term in patients undergoing major non-cardiac surgery. Despite that, the overall cost-effectiveness of this approach compared to the widely used Revised Cardiac Risk Index (RCRI) remains uncertain. A cost–consequence analysis from the VISION group found that cTn monitoring resulted in moderate additional costs, but it may offer value in detecting myocardial injury in high-risk patients. Some studies have explored strategies to reduce costs by selectively applying troponin testing to those with higher baseline risks, though the long-term cost-effectiveness of these strategies is still not well established [9]. The Canadian Cardiovascular Society guidelines recommend restricting biomarker use to patients with a baseline risk of over 5%, which further complicates the evaluation of its cost-effectiveness in broader patient populations. Therefore, while cTn testing holds potential for identifying high-risk individuals, further research is needed to determine its definitive cost-effectiveness relative to RCRI in clinical practice.

## 6. Future Directions and Research Gaps

### 6.1. Personalised Risk Stratification and Treatment Planning

Cardiac troponin continues to have a significant impact on clinical practice, particularly in enhancing perioperative risk stratification. Its integration into care pathways enables personalised perioperative management, such as optimising the use of medications like statins and antiplatelets [50,55]. Identifying patients who may benefit from targeted monitoring allows for more judicious use of healthcare resources, potentially lowering costs and improving patient outcomes [12].

**Figure 2:**
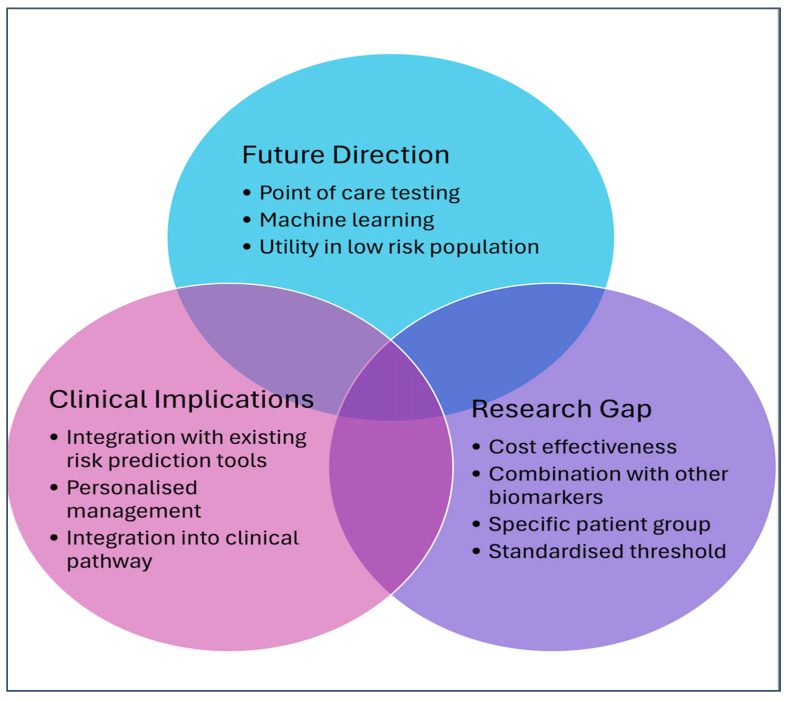
A conceptual diagram of the relationship between future directions, research gaps, and the clinical implications of cardiac troponin in a perioperative setting.

Emerging evidence supports the combination of NT-proBNP and high-sensitivity cardiac troponin (hs-cTn) as a more robust approach to risk assessment. Preoperative elevations in NT-proBNP and cTnT are strongly associated with increased mortality and cardiovascular complications, including myocardial infarction and heart failure, in surgical patients [4]. Incorporating both biomarkers may improve diagnostic accuracy and guide prevention strategies for perioperative myocardial injury. Figure 2 illustrate a conceptual diagram of the relationship between future directions, research gaps, and the clinical implications of cardiac troponin in a perioperative setting

### 6.2. Patient-Specific Biomarker Thresholds

The significance of hs-cTn levels can vary depending on individual patient characteristics such as age, gender, comorbidities, and baseline biomarker levels. Standardised thresholds that ignore these factors may lead to misclassification. There is a pressing need to personalise hs-cTn interpretation using adjusted cut-offs tailored to specific subgroups [52,53]. Further research is needed to establish standardised protocols that integrate these variables to improve the sensitivity and specificity of myocardial injury detection in the perioperative setting.

The utility of high-sensitivity cardiac troponin (hs-cTn) in a diverse surgical population, including low-risk patients, necessitates further validation through large-scale prospective cohort studies. These studies should aim to assess the predictive value of hs-cTn across different surgical risk profiles, identifying its role in guiding perioperative decision making and improving patient outcomes.

### 6.3. Economic Evaluations of Routine hs-cTn Monitoring

Additionally, to determine the overall cost-effectiveness of routine perioperative hs-cTn monitoring, a comprehensive clinical study should be conducted alongside a thorough economic evaluation. This evaluation should consider factors such as healthcare resource utilization, hospital length of stay, potential reductions in postoperative complications, and overall cost savings resulting from early intervention and improved risk stratification. By integrating both clinical and economic perspectives, future research can provide a more comprehensive understanding of the benefits and feasibility of implementing routine hs-cTn monitoring in perioperative care.

### 6.4. Integration of Artificial Intelligence and Predictive Modelling

Machine learning presents a novel opportunity to enhance cardiac risk prediction by integrating hs-cTn data with clinical variables [56]. Machine learning methods have been utilised to enhance the predicted accuracy of high-sensitivity cardiac troponin for cardiac risk evaluation. Models like extreme gradient boosting (XGBoost) have been employed to detect high-risk patients for myocardial injury post-surgery, exhibiting remarkable prediction accuracy, with area under the curve (AUC) values surpassing 0.95 [57]. These models incorporate diverse clinical inputs such as age, comorbidities, and surgical type, which have shown superior performance compared to conventional scores like the Revised Cardiac Risk Index (RCRI).

Furthermore, adopting machine learning methodologies that develop prediction models by integrating biomarkers with clinical data is essential for advancing risk stratification and perioperative decision making. Big data analytics has enabled the creation of comprehensive and dynamic risk models, with promising performance metrics and real-time applicability. Future research should focus on refining these models, validating them across diverse populations, and evaluating their feasibility for clinical use.

## 7. Conclusions

Cardiac troponin, particularly highly sensitive cardiac troponin, is an effective predictive marker for major adverse cardiovascular events and mortality in the perioperative setting. It facilitates risk stratification and supports timely interventions to improve patient outcomes. Therefore, further research is essential to establish evidence-based protocols incorporating hs-cTn into clinical practice and thus enhancing perioperative care.

## Figures and Tables

**Table 1:** Possible causes of raised cardiac troponin other than coronary occlusion in the perioperative period [26,27].

Preoperative	Intraoperative	Postoperative
Heart failure	Surgical stress response	Sepsis and septic shock
Cardiomyopathies	Cardiac arrhythmias	Acute pulmonary embolism
Drug-induced direct cardiotoxic injury (antitumour chemotherapy, methamphetamines)	Massive haemorrhage	Acute heart failure
Chronic kidney disease		Acute kidney Injury
Pre-existing pulmonary embolism		

**Table 2:** Correlation of peak postoperative hsTnT thresholds from the first 3 days after surgery and mortality rate [9].

Postoperative hs-cTnT	Mortality Rate
<20 ng/L	<0.5%
20–65 ng/L	3.0%
65–999 ng/L	9.1%
>1000 ng/L	29.6%

**Table 3:** List of relevant studies that have examined the prognostic value of troponin in the prediction of MACEs [3,15,16,33,34,35,36,37,38].

No	Title	Year	Sample Size	Author	Key Findings	Type of Study
1	Postoperative elevated cardiac troponin levels predict all-cause mortality and major adverse cardiovascular events following noncardiac surgery: a dose–response meta-analysis of prospective studies	2023	53,518	Lijing Yang et al. [33]	Elevated postoperative troponin is associated with MACE in a dose–response manner	Dose–response meta-analysis
2	Prognostic performance of preoperative cardiac troponin for the prediction of major adverse cardiac events and mortality in noncardiac surgery: a systematic review and meta-analysis	2019	13,386	Caroline A. S. Humble et al. [16]	Preoperative cTn and perioperative change in cTn might be valuable predictors of MACE and/or all-cause mortality in adult noncardiac surgical patients; its overall prognostic performance remains uncertain	Systematic review and meta-analysis
3	Troponin elevations after non-cardiac surgeryare predictive of major adverse cardiac events and mortality: a systematic review and meta-analysis	2016	2193	Sarah Ekeloef et al. [15]	Post-op troponin elevation is an independent predictor of major adverse cardiac events and mortality within 30 days and 1 yr after non-cardiac surgery	Systematic review and meta-analysis
4	Meta-analysis of preoperative high-sensitivity cardiac troponin measurement in non-cardiac surgical patients at risk of cardiovascular complications	2020	4836	Bing-Cheng Zhao et al. [34]	Raised preoperative high-sensitivity troponin levels are associated with a higher risk of short-term major adverse cardiac events, short-term mortality and long-term mortality	Meta-analysis of cohort studies
5	Evaluation of perioperative highly sensitive cardiac troponin i as a predictive biomarker of major adverse cardiovascular events after noncardiac surgery	2020	93	Alejandro Millán-Figueroa et al. [35]	Preoperative hs-cTnI is an independent predictice risk factor for MACE at 30 days and 1 year after non-cardiac surgery and for all-cause of mortality at 1 year after non-cardiac surgery	Prospective cohort design
6	High-sensitivity Troponin I Predicts Major Cardiovascular Events after Non-Cardiac Surgery: A Vascular Events in Non-Cardiac Surgery Patients Cohort Evaluation (VISION) Substudy	2023	4553	Flávia Kessler Borges et al. [36]	A peak hsTnI level of ≥75 ng/L was linked to a more than 5-fold increase in the risk of MACE compared to levels < 75 ng/L, with adjusted hazard ratios of 4.53 for levels between 75 ng/L and <1000 ng/L and 16.17 for levels ≥ 1000 ng/L, indicating that this threshold could be utilized for diagnosing myocardial injury after non-cardiac surgery	Prospective cohort design
7	Incidence of major adverse cardiac events following non-cardiac surgery	2021	2265	Lorraine Sazgary et al. [3]	Patients with postoperative myocardial injury as evidenced by raised hs-cTnT are at increased risk of death within 1 year of MACE	Prospective observational study
8	Cardiac Biomarkers Predicting MACE in Patients Undergoing Noncardiac Surgery: A Meta-Analysis	2019	7877	Lijun Zhang et al. [37]	Elevations of BNP/NT-proBNP, cTnI/cTnT, and hs-CRP, immediately preoperation or postoperation, can predict much higher risk of postoperative MACE in patients undergoing noncardiac surgery	A Meta-analysis
9	Association of preoperative troponin levels with major adverse cardiac events and mortality after noncardiac surgery: a systematic review and meta-analysis	2018	10,371	Jian-Tong Shen et al. [38]	Preoperative high troponin levels are significantly associated with adverse cardiac events and mortality after noncardiac surgery	Systematic review and meta-analysis of observational studies

## Data Availability

Not applicable.
